# An Integrated GPR B-Scan Preprocessing Model Based on Image Enhancement for Detecting Subsurface Pipes

**DOI:** 10.3390/s25237202

**Published:** 2025-11-25

**Authors:** Zhengyi Shi, Fanruo Li, Hanchao Ma, Hong Huang, Le Wu, Maohua Zhong

**Affiliations:** 1 School of Safety Science, Tsinghua University, Beijing 100084, China; shizy21@mails.tsinghua.edu.cn (Z.S.);; 2 School of Artificial Intelligence, China University of Mining and Technology, Beijing 100083, China

**Keywords:** GPR B-scan, subsurface pipe, image enhancement, thresholding, hyperbola segmentation

## Abstract

Ground-penetrating radar (GPR) has been proven effective for detecting subsurface pipes in a nondestructive way, typically with manual processing and decision-making. However, existing automatic models for segmenting the target hyperbolas often lack generalization across different pipe radii, varying subsurface media, and complex field conditions. This is especially reflected in B-scans with diverse or small-scale hyperbolas, often accompanied by cluttered and irregular noise. In this paper, an automatic preprocessing model is proposed to enhance the interpretation of B-scans under challenging conditions. The model includes a ground reflection removal algorithm (GRRA), the data gravitational force enhancement (DGFE) method, and a global–local thresholding technique consisting of dilation-based local thresholding and segmentation (DLTS). First, a frequency-domain filter based on the fast Fourier transform and a spatial filter are applied to the raw B-scan to remove obstructive ground reflection strips. Owing to the minimal intensity differences among the target hyperbola, multiples, and background, the DGFE approach is introduced to amplify the main body of the hyperbola, distinguishing it from the noise. Finally, the target hyperbola is extracted from the grayscale image by an integrated thresholding approach. The approach initially employs global thresholding to eliminate all information except for part of the hyperbola, followed by DLTS, which uses a dilation operation with local thresholding to fully segment the hyperbola. The proposed model is evaluated on two distinct datasets and compared with several state-of-the-art methods. The results demonstrate its effectiveness, particularly in terms of cross-dataset generalization.

## 1. Introduction

In recent years, the assessment of subsurface pipe conditions in urban areas has gained the attention of decision-makers and researchers because of the growing need for accurate data on buried infrastructure and operational monitoring systems [1,2,3,4]. Multiple nondestructive detection tools are commonly used, with ground-penetrating radar (GPR) standing out for its portability, flexibility, and ability to detect both metallic and nonmetallic objects [5]. GPR operates on the basis of electromagnetic wave principles, including wave propagation through a medium and reflection at the interface between different media. These principles impose certain limitations on its application. First, there must be a significant difference in permittivity or dielectric constant between the target object and the surrounding medium. This difference is crucial for generating electromagnetic reflection that is strong enough to be distinguishable from the background. Second, the target object must be relatively shallow, or the surrounding medium must be uniform. Otherwise, electromagnetic wave attenuation and scattering can significantly impair detection accuracy [6].

When the GPR operates, the device moves along a survey line on the ground, and samples are taken at regular intervals. At each sampling point, the GPR records a time-response signal corresponding to a specific electric or magnetic field component over the duration of the scan. This time response is referred to as the A-scan at that trace. As the GPR moves along the survey line, an A-scan is collected at each trace. By arranging these A-scans sequentially in the direction of motion, a two-dimensional array is formed, where each pixel value corresponds to a specific position–time coordinate. This array is referred to as the B-scan of the survey line. In general, although the horizontal and vertical axes of a visualized B-scan carry physical meanings, they do not directly represent continuous physical quantities. The spacing between ticks along the horizontal axis typically corresponds to the spatial sampling interval of the GPR device, or the trace interval, while that along the vertical axis corresponds to the temporal sampling interval. Therefore, without any scaling applied, each pixel in the image represents the signal on a certain trace in the horizontal direction and a certain sample in the vertical direction. The visualized B-scan in this paper adopts this convention, unless otherwise declared. When the survey line is oriented perpendicular to the axis of a subsurface pipe, its B-scan typically exhibits a hyperbolic signature. This hyperbola contains information about the outer radius of the pipe, the position of its projection onto the ground surface, its burial depth, and the average velocity of electromagnetic waves through the surrounding medium [7]. However, in complex subsurface environments, hyperbolas may overlap, be obscured by noise, or have artifacts brought by multiple reflections. Therefore, hyperbola segmentation from the original B-scan image becomes essential for accurate quantitative analysis. By extracting the target hyperbola, the influence of background noise and multiple reflections can be effectively reduced, and we can focus more on the most informative portions of the signal, thereby improving the accuracy of parameter estimation, such as pipe radii and the electromagnetic wave velocity under specific medium conditions. Consequently, hyperbola segmentation serves as a fundamental preprocessing step for reliable information extraction and subsequent analysis. In this paper, the collection of procedures required for hyperbola segmentation is referred to as the preprocessing operation.

Preprocessing methods based on convolutional neural networks (CNNs), including deep CNNs [8], faster region-based CNNs [9], and regional CNNs [10], are widely used because of their effectiveness in processing images. In most cases, a combination of field and synthetic data is employed to ensure sufficient data quality. When data are insufficient, techniques such as generative adversarial networks (GANs) [11] and transfer learning [12] are introduced. These comprehensive methods typically achieve accurate and refined preprocessing results, but their performance depends heavily on the amount and quality of available data. Preprocessing involves multiple steps, including denoising, background removal, and hyperbola segmentation. Noise is common in field GPR data, and its origins are often complex. Traditional denoising methods such as empirical mode decomposition (EMD) [13] and the shearlet transform [14] are used, as well as improved techniques, including the nonsubsampled shearlet transform (NSST), block-matching and 3D filtering (BM3D) [15], and two-dimensional variational mode decomposition (2D-VMD) [16] integration. The ground reflection, an important type of background, which appears as alternating black and white horizontal strips, results from electromagnetic waves reflecting off the ground surface. These strips can intersect with hyperbolas and must be removed before further processing. Various mean subtraction filters based on the running average filter are used for this purpose  [17,18,19]. The running-average-based filters work well with high-resolution images or when the hyperbola’s tails extend sufficiently. Other techniques, such as Fourier frequency-domain filters  [20], are also employed. To segment the desired hyperbola from the image, researchers often convert the image into a binary format through a process known as thresholding. Global thresholding methods, including techniques such as Otsu’s method [21], the method that accounts for the longitudinal gradient [18], and adaptive methods [22], are popular because of their computational efficiency. The final step is hyperbola segmentation. Effective methods in this stage include column-connection clustering (C3) [17], the open-scan clustering algorithm (OSCA) [18], and column-based transverse filter points (CTFP) [9], all of which have been proven successful under various conditions.

For the whole preprocessing flow, a method that preprocesses GPR images, applies a Fourier transform for frequency-domain analysis, and uses local binary pattern (LBP) analysis to emphasize hyperbolic peaks is proposed [23], ultimately improving feature recognition in GPR data. An integrated method adopting edge detection and point cloud segmentation is also developed to localize subsurface pipes from GPR images [24]. The approach demonstrates the effectiveness of frequency-domain transformations and texture analysis in enhancing GPR image interpretation. Ref.  [25] proposes a method to detect and extract hyperbolas in GPR images using wavelet analyses and Gabor filtration, and then an analytic hierarchy process is used to classify the filtered images into categories of certainty and uncertainty. This study also demonstrates the effectiveness of processing GPR signals in the frequency domain. In addition, it proposes a two-stage approach that first focuses primarily on the radiometric characteristics of the data and then considers the geometric relationships among targets. This provides an important reference for subsequent studies that address the problem sequentially based on signal-intensity features and topological characteristics.

However, limitations exist in current algorithms, particularly regarding the trade-off between generalization ability and processing speed. In practical GPR detection scenarios, such as untested sites where solid and pipe properties differ significantly from existing data, many machine learning approaches have become ineffective. Non-machine learning methods may perform well individually but struggle when integrated into a complete pipeline. Additionally, algorithms proposed by different researchers can sometimes conflict. As mentioned earlier, field conditions can further degrade B-scan quality, resulting in fluctuating ground reflection strips, dominant clutter, indistinct hyperbola tails, or faint hyperbola bodies. This highlights the need for improvements to existing algorithms and the development of novel approaches.

**Figure 1:**
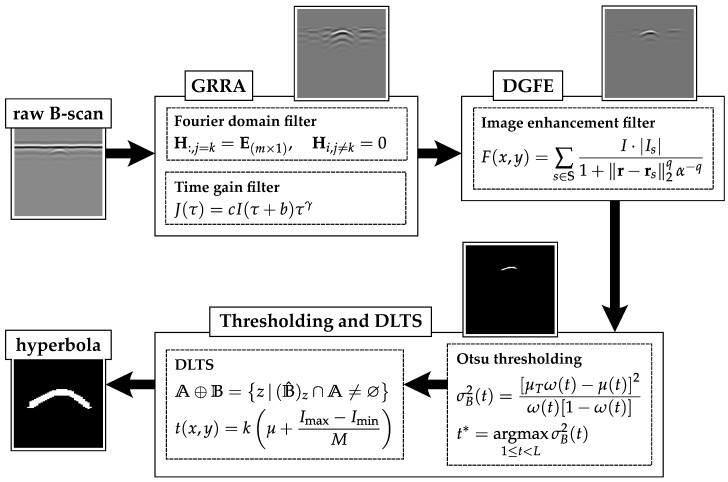
Framework of the proposed workflow, which includes a ground reflection removal algorithm (GRRA), an image enhancement filter (DGFE), and a global–local thresholding technique centered on DLTS.

To address these challenges, this paper presents an integrated model to preprocess B-scans; the proposed model is effective across various datasets acquired from conditions where pipe radii vary, burying media and pavement properties may be homogeneous, and the field conditions are complex. As shown in Figure 1, the model consists of (a) a ground reflection removal algorithm (GRRA); (b) a critical data gravitational force enhancement (DGFE) model to enhance the data array by emphasizing the target hyperbola while suppressing multiples and background noise; and (c) a global–local thresholding technique that combines dilation-based local thresholding and segmentation (DLTS) to simultaneously achieve local thresholding and hyperbola segmentation. The work in this paper can help improve GPR processing systems by enhancing generalizations across various detection scenarios and increasing detection efficiency. These improvements can support field data collection for pipe construction projects and provide valuable insights for decision-makers.

The rest of this paper is organized as follows. In Section 2, the GPR B-scan is analyzed in the Fourier domain, and the GRRA method is described. Section 3 introduces the techniques of data enhancement (DGFE), thresholding, and segmentation (DLTS). In Section 4, the proposed model is applied to two real-world datasets together with other methods to test its effectiveness and efficiency. Finally, in Section 5, the value of this work and some future expectations are discussed.

## 2. Ground Reflection Removal

After an electromagnetic wave is emitted from the source antenna, it propagates through the air and reflects off the ground surface, and the reflected wave is received by the receiving antenna. In the B-scan, ground reflections typically manifest as alternating strips of fixed width, oscillating between negative and positive values. These ground reflection strips can interfere with the interpretation of underground pipe hyperbolas, as they often intersect with hyperbolic features, making it challenging to extract meaningful information from the raw signal. In fact, ground reflections significantly impact the subsequent processing steps. Apart from the direct airwave signal, the ground reflection is usually the first strong signal detected by the receiving antenna. Given that pipelines are typically buried tens of centimeters to several meters underground, the reflections from the medium–pipeline interface are often substantially attenuated compared to ground reflections. Furthermore, subsequent processing steps in this paper, such as data enhancement and thresholding, consider signal strength as the primary variable, despite accounting for data point correlations. Therefore, if ground reflections are not effectively removed in the first step, their relatively high intensity and spatial overlap with hyperbolas may cause them to be erroneously treated as useful signals, leading to misinterpretation and potential failure of the entire preprocessing workflow. Consequently, ground reflection removal is a crucial preprocessing step to ensure accurate hyperbola extraction and subsequent analysis.

To remove the ground reflections, the ground reflection removal algorithm (GRRA) is proposed, whose main component is a Fourier domain filter. This section includes an analysis of the Fourier spectrum characteristics of B-scans and the algorithm deployment based on these characteristics.

### 2.1. DFT Analysis

The Fourier transform is particularly effective at handling periodic signals. In this case, the two-dimensional discrete Fourier transform (DFT) is applied, written as

(1) $$\hat{I}\left(\hat{x},\hat{y}\right)=\sum_{x=0}^{M-1}\sum_{y=0}^{N-1}I\left(x,y\right)\exp\left[-j2\pi \left(\frac{\hat{x}x}{M}+\frac{\hat{y}y}{N}\right)\right],$$

where *I* denotes the B-scan; *x* and *y* represent discrete coordinates in the B-scan, with the origin at the top-left corner; *M* and *N* denote the width and height of the B-scan, respectively; the term j refers to the imaginary unit; and $\hat{\cdot }$ indicates a Fourier domain variable. Specifically, $\hat{x}=0,1,2,\ldots ,M-1$, and $\hat{y}=0,1,2,\ldots ,N-1$. $\hat{I}$ is a discrete complex function, and its magnitude $|\hat{I}\left(\hat{x},\hat{y}\right)|$, shown in a Cartesian plane indexed by $\left(\hat{x},\hat{y}\right)$, is the Fourier spectrum of *I*. In practice, the spectrum of I(x,y) is actually that of ${\left(-1\right)}^{x+y}I\left(x,y\right)$, which places $\hat{x}=0$ and $\hat{y}=0$ at the origin of the spectrum. For the remainder of this paper, the term *spectrum of* I(x,y) refers to the spectrum of ${\left(-1\right)}^{x+y}I\left(x,y\right)$.

In the time domain of a B-scan, in addition to noises, there are two major components: ground reflection strips and one or more hyperbolas, often accompanied by multiples beneath them. Both components appear distinctly in the Fourier spectrum. The ground reflection consists of a series of horizontal lines in the time domain. Since any straight line at a specific inclination will, in the spectrum, transform into another straight line that passes through the origin at a complementary inclination, the ground reflection becomes a series of vertical lines at $\hat{x}=0$. Furthermore, the periodicity and magnitude of its Fourier transform for each strip vary depending on its vertical position and energy. As a result, the vertical lines in the spectrum change in value along the $\hat{y}$ direction.

**Figure 2:**
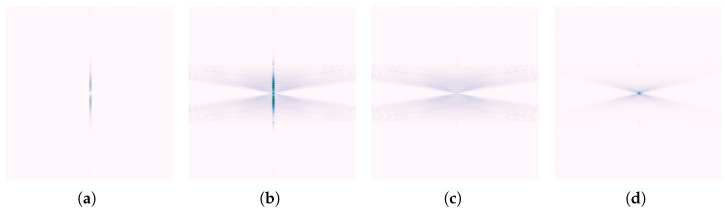
Spectra of various simulated B-scans, where blue indicates a higher magnitude than pink. (**a**) Spectrum of ground reflection strips without hyperbolas; (**b**) spectrum of ground reflection strips with a hyperbola and its multiple reflections; (**c**) spectrum of a hyperbola without ground reflection strips; (**d**) spectrum of a hyperbola without multiple reflections beneath it.

Figure 2 illustrates the spectral characteristics of simulated B-scans under different conditions: with and without ground reflection strips, and with and without hyperbolic targets. From Figure 2a, it can be observed that the spectrum of ground reflection strips appears as a narrow vertical line at the *x* DC component. Along this line, the magnitude increases from $\hat{y}=0$ and then gradually decreases. In contrast, a hyperbola is characterized by two inclined lines passing through the origin. These lines exhibit faint shadow regions in $\pm \hat{y}$ directions, and the presence of multiple reflection artifacts increases the magnitude in the shadow region.

**Figure 3:**
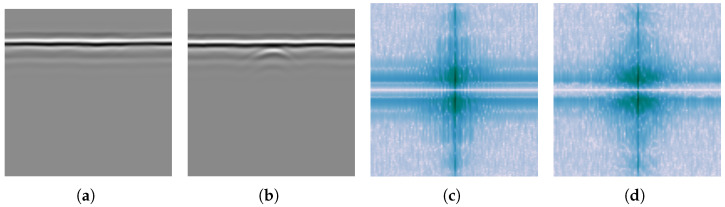
B-scans from a field experiment and their corresponding spectra, where blue indicates a higher magnitude than pink. (**a**) B-scan with no pipe buried; (**b**) B-scan with a ductile iron pipe buried at a depth of 10cm; (**c**) logarithmic spectrum of (**a**); (**d**) logarithmic spectrum of (**b**).

Spectra from B-scans obtained through field experiments are expected to exhibit similar properties. Figure 3 shows the logarithmic spectra of two B-scans under different experimental conditions: with and without a buried pipe. It can be observed that in the presence of buried pipelines, the B-scan spectrum also exhibits a pattern similar to that in Figure 2b near the DC component. Two less prominent lines pass through the origin, with a broad shadowed region enclosed (closer to the $\hat{y}$-axis) between them.

**Figure 4:**
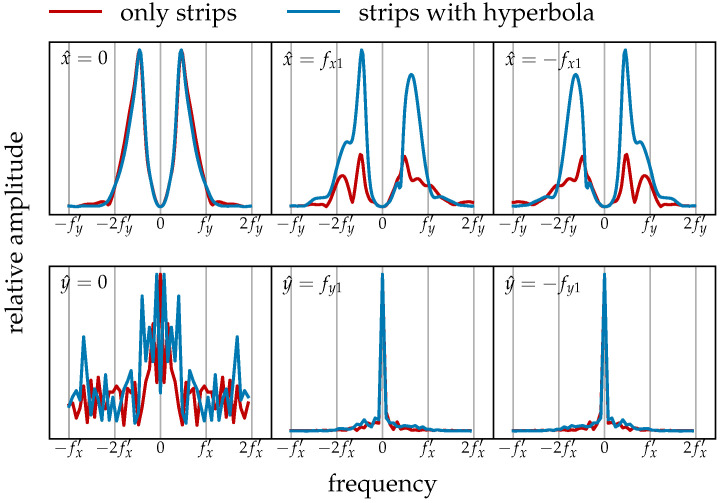
Cross-sections at different $\hat{x}$s and $\hat{y}$s in the spectra of Figure 3a,b. The horizontal axis represents the frequency along the $\hat{y}$ or $\hat{x}$ direction, where $f_{y}^{\prime }$ and $f_{x}^{\prime }$ are a quarter of the Nyquist frequency in $\hat{y}$ and $\hat{x}$ directions, respectively; the vertical axis represents the magnitude.

The differences between the spectral characteristics of ground reflection strips and the hyperbola suggest that, to remove the ground reflection strips while retaining the hyperbola, a Fourier domain filter can be applied. To explore this further, curves that describe the magnitude’s variation with frequency are shown in Figure 4. For example, the notation $\hat{x}=0$ in the top-left corner indicates the change along the line $\hat{x}=0$ in the spectrum plane. When there is a significant contrast between the red and blue curves in the same plot, careful attention is needed, as these regions likely contain important information about the hyperbola. Conversely, frequency components in other regions can largely be removed. As indicated in Figure 4, the *x* DC component should be suppressed. The design of this filter will be further discussed in the next section.

### 2.2. Removal Algorithm

This section introduces GRRA, which comprises a frequency-domain filter to damp the ground reflection strips and a spatial filter. Since the most prominent feature in the spectrum of a B-scan including ground reflection strips is a narrow vertical bright line, it is intuitive to apply a straightforward filter. The transfer matrix H of this filter is written as

(2) $$H_{:,j=k}=E_{\left(m\times 1\right)},\;H_{i,j\neq k}=0,$$

where *k* denotes the column index, $\hat{x}=0$, the subscript :,j=k refers to the *k*th column of the matrix, and E is a matrix of ones. The filter H sets all the components at $\hat{x}=0$ to zero while leaving all the other components unchanged. The transfer function is then applied to the raw B-scan as

(3) J=H⊙I,

where J and I represent the processed and raw arrays, respectively, and where ⊙ refers to the Hadamard product (elementwise multiplication) operator. Compared to another frequency-domain filter, reported by [20],

(4) $$g\left(\hat{x},\hat{y}\right)=1-\exp\left(-\frac{{\hat{y}}^{2}}{2\sigma^{2}}\right),$$

where σ is a control constant to determine the width of the filter, Equation (3) slightly reduces the computational cost and features a narrower response function. This makes it more suitable for addressing the spectral characteristics of ground reflections, which are typically concentrated within a very narrow band in the spectrum. Even when the ground surface in the data acquisition environment exhibits slight undulations, the spectral spread of ground reflection strips remains limited. A steep filter is sufficient to suppress the ground reflection band and better preserves useful information outside of it. A cross-dataset validation of Equation (3) will be presented in a later section.

**Figure 5:**
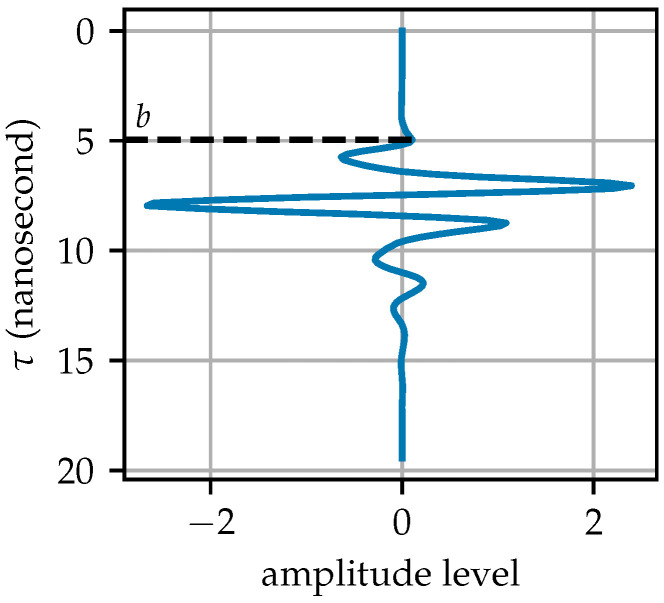
Determination of the cutoff position in the A-scan. The filter identifies the first local maximum value of the electromagnetic wave amplitude, which corresponds to a specific position *b* on the time axis. In this A-scan, the portion where τ<b should be removed to align the first ground reflection signal properly.

In addition, the hyperbola is plotted with deeper signals that the GPR receives later than the signals reflected off the ground surface. These deeper signals are less intense. As a result, enhancing the deeper signal can highlight the hyperbola and help with the removal process. This paper adopts a spatial filter known as gamma transformation, which is applied as a time gain filter in the form of

(5) $$J\left(\tau \right)=cI\left(\tau +b\right)\tau^{\gamma },$$

where *J* and *I* represent the processed and input arrays, respectively; τ is the round-trip time from the emission to the receiving of the electromagnetic wave; *b* is the time bias constant or the cutoff position; and *c* and γ are control constants, typically with c=1. When γ>1, the signal is amplified to a greater extent, as it penetrates deeper. The bias constant *b* is determined by identifying the first local maximum in the A-scan (marked in Figure 5 by a horizontal black dashed line). This ensures that the filter is applied after the first ground reflection strip, as indicated by y+b, starting from the point where the GPR receives the first signal pulse.

**Figure 6:**
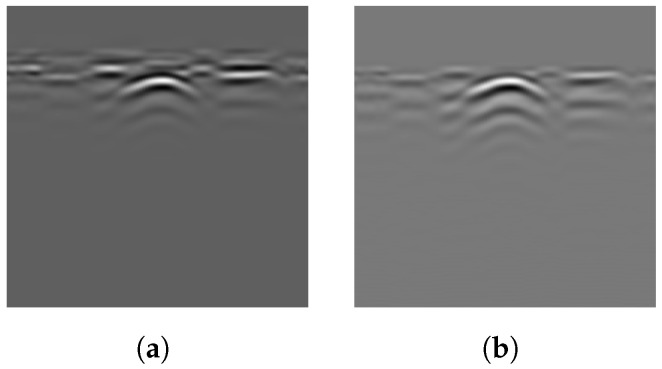
Performance of GRRA: (**a**) A visualized array filtered by the frequency filter, where the ground reflection strips are largely removed; (**b**) a visualized array filtered by the time gain filter, which further weakens the remaining ground reflection strips and enhances the hyperbola.

The arrays processed using Equations (3) and (5) are shown in Figure 6. Most of the ground reflection strips have been removed, with only a few remaining, which have been significantly damped by the gamma-transformation filter. The target hyperbola is now more distinct from the background due to GRRA, facilitating subsequent steps in extracting the hyperbola from the array.

## 3. Thresholding and Segmentation

Before the parameters that define the shape of the hyperbola are analyzed, it is essential to extract it from the background. Various thresholding methods exist that perform a binarization operation, mapping each data point of the B-scan to either a black or a white pixel. In terms of a B-scan of a pipe primarily made of metal, the main hyperbola appears brighter than its multiples and the background. The ideal thresholding result would map the hyperbola to white while turning everywhere else black. However, traditional, commonly used global thresholding methods struggle when the main hyperbola is only slightly brighter than its multiples. In these cases, the method may preserve the main hyperbola but also retain some of its multiple reflection artifacts. This occurs because deeper signals have lower grayscale levels, such that the grayscale level at the tail of the main hyperbola can be lower than that at the head of its artifacts.

Generally, there are two approaches to addressing the varying requirements for threshold selection. One option is to enhance the region of interest before applying global thresholding, whereas the other option is to use local thresholding despite its computational complexity. To balance preservation of useful information, speed, and hyperbolic shape, a combination of data enhancement, global thresholding, and local thresholding is adopted. First, a novel method named data gravitational force enhancement (DGFE) is proposed to intensify the grayscale within the hyperbola region. Next, Otsu’s global thresholding method is applied to determine a global threshold that sufficiently extracts the main part of the target hyperbola. Finally, the tail of the extracted hyperbola head is reattached through a local thresholding technique, dilation-based local-thresholding segmentation (DLTS), which is applied only around the edge of the head.

### 3.1. DGFE

To the human eye, the main hyperbola is bright enough to be distinguished from its artifacts because the grayscale difference between the hyperbola and the background is more significant than that of its artifacts. The intense sections of the hyperbola seem to accumulate, as if they were being repelled by the less intense part of the array. On the basis of this observation, this paper proposes an enhancement method that amplifies the significance of pixel clusters, emphasizing the main hyperbola while differentiating it from its artifacts and the background noise. The proposed method draws inspiration from the principles of gravitational theory, a fundamental concept in physics, and is therefore named data gravitational force enhancement.

As the name suggests, DGFE draws an analogy between individual pixels and static mass points in a vacuum. Li was the first to conceptualize pixels as particles with masses that interact with each other [26]. On the basis of Li’s work, Wu introduced a data field model to identify the transition region between an object and the background [3], employing theories from nuclear, gravitational, and electrostatic fields. This paper modifies Wu’s gravitational model to increase its applicability to B-scan data. In Wu’s gravitational model, the gravitational potential φ generated by the pixel at (x,y) at another point $\left(x^{\prime },y^{\prime }\right)$ is calculated as

(6) $$\varphi_{x,y}\left(x^{\prime },y^{\prime }\right)=\frac{Gm_{x,y}}{1+{∥r-r^{\prime }∥}_{2}^{q}\alpha^{-q}},$$

where *G* is the gravitational constant; *m* denotes the mass; r denotes the position vector; the operator ${∥\cdot ∥}_{2}$ refers to the 2-norm, commonly the Euclidean norm; and α and *q* are control constants. The data field at the position of a pixel is the scalar sum of the potentials generated by all other pixels, and its gradient can indicate the location of transition regions. In this way, the data field can identify prominent edges located near transition regions across the entire image, enabling edge detection and image segmentation. It is worth noting that the data field is a global concept. At each point in the image, the data field is computed as the scalar sum of contributions from all other points. However, in the context of B-scan analysis, where hyperbola extraction is the primary objective, local contextual information is far more important than global information. This is because the goal is to distinguish hyperbola signals from background signals within the neighborhood of a hyperbola candidate, rather than across the entire B-scan. Therefore, to adapt the data field approach to the specific task, it is reasonable to localize the computation by focusing only on the proximity of each pixel.

In a B-scan array, the data value is proportional to the electromagnetic component at a specific sample and trace. The main hyperbola, visible to the human eye, contains only positive field strength values, with the negative values generated by the emission pulse appearing directly beneath it. A metaphorical hypothesis is proposed in which positive data points are attracted by other positive points and repelled by negative points, whereas negative points are repelled by all other points. This interaction amplifies the clustering behavior of positive points, particularly in forming the main hyperbola, and distinguishes it from others. The net data gravitational force *F* on a data point (x,y) is defined as

(7) $$F\left(x,y\right)=\sum_{s\in S}\frac{I\cdot |I_{s}|}{1+{∥r-r_{s}∥}_{2}^{q}\alpha^{-q}},$$

where S is the set of all points in the proximity determined by a two-dimensional squared window of a certain size; *I* denotes the signed data value. In this paper, q=2 and α=1, and the selection of window size will be further discussed. The original data field approach illustrated by Equation (6) computes the data field generated by all pixels in the image at each point, where the distribution of the data field reflects the positional relationship between local and global information. However, in a B-scan containing regions of interest, incorporating global information may dilute crucial local contextual cues. Therefore, DGFE focuses more on the proximity of a point, that is, the information within a window of a predefined size. Intuitively, the key to the problem lies in whether a hyperbola can be distinguished from the background, while the global content outside the local region is largely trivial. Secondly, DGFE is not fully focused on the data field itself. Instead, we introduce a new definition of net gravitational force, which explicitly takes into account the value of the pixel at (x,y). While Wu’s method uses the absolute difference between two pixel values in the numerator, DGFE replaces this with the product of the two pixel values. This modification further amplifies the contrast between high-intensity and low-intensity regions in the B-scan. Additionally, a key modification is made. The pixel at (x,y) retains its signed value, while all other pixels within the window use their absolute values. The rationale is that the radar’s transmitted and received signal is a pulse, and the electric field strength in the *z* polarization direction contains both positive and negative components, which is meaningful in B-scan interpretation. Therefore, we aimed to incorporate the signal polarity into the computation. This produces an interesting effect. The more surrounding positive or negative pixels a positive pixel has, the stronger the positive net force it experiences. In contrast, negative pixels are influenced only by negative net forces. In other words, positive pixels are attracted by all nearby pixels, while negative pixels are effectively repelled by everything. As a result, clusters of positive pixels are further enhanced, while negative pixels are suppressed.

**Figure 7:**
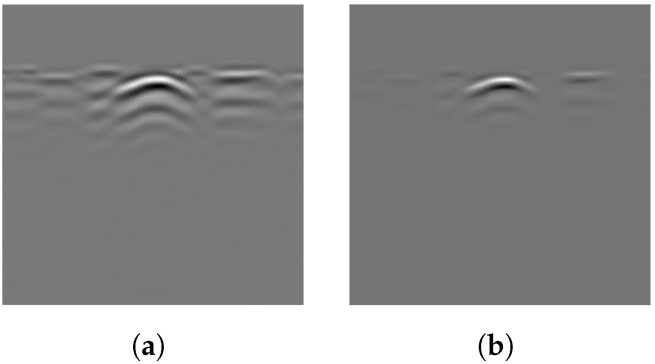
Result of DGFE with a window size of 5: (**a**) The input array, newly processed with the time gain filter; (**b**) the enhanced array, showing a visualization of the force field, where the main hyperbola is dominant.

A larger *F* indicates that the data point is more likely part of a cluster of positive values, distinguishing it from the surrounding area. Conversely, a smaller *F* indicates a region of negative values or positive points with lower intensity. After the force on each data point in a B-scan is calculated, the force field can be visualized. In the force field shown in Figure 7b, the net force on the points of the main hyperbola is clearly larger than that on the background. Visually, the main hyperbola is successfully distinguished from the background, significantly aiding the subsequent thresholding step.

### 3.2. Global Thresholding

Otsu’s thresholding method is a fast and reliable unsupervised algorithm that performs well in determining a global threshold in various situations [27]. It works by finding the integer grayscale level $t^{*}$ that maximizes the interclass variance $\sigma_{B}^{2}\left(t\right)$ between two classes split by $t^{*}$, which is given by

(8) $$\begin{array}{c} t^{*}=\underset{1\leq t<L}{\mathrm{argmax}}\sigma_{B}^{2}\left(t\right)=\underset{1\leq t<L}{\mathrm{argmax}}\frac{{\left[\mu_{T}\omega \left(t\right)-\mu \left(t\right)\right]}^{2}}{\omega (t)[1-\omega (t)]}, \end{array}$$

where *L* is the maximum grayscale level, and in this paper, L=255; ω(t) and μ(t) are derived from

(9) $$\begin{array}{cc} \; & \mu_{0}=\sum_{i=1}^{t}\frac{ip_{i}}{\omega_{0}}=\frac{\mu (t)}{\omega (t)},\\ \; & \mu_{1}=\sum_{i=t+1}^{L}\frac{ip_{i}}{\omega_{1}}=\frac{\mu_{T}-\mu \left(t\right)}{1-\omega (t)}, \end{array}$$

and

(10) $$\begin{array}{cc} \; & \omega_{0}=\sum_{i=1}^{t}p_{i}=\omega \left(t\right),\\ \; & \omega_{1}=\sum_{i=t+1}^{L}p_{i}, \end{array}$$

and $\mu_{T}$ is written as

(11) $$\mu_{T}=\sum_{i=1}^{L}ip_{i}.$$

**Figure 8:**
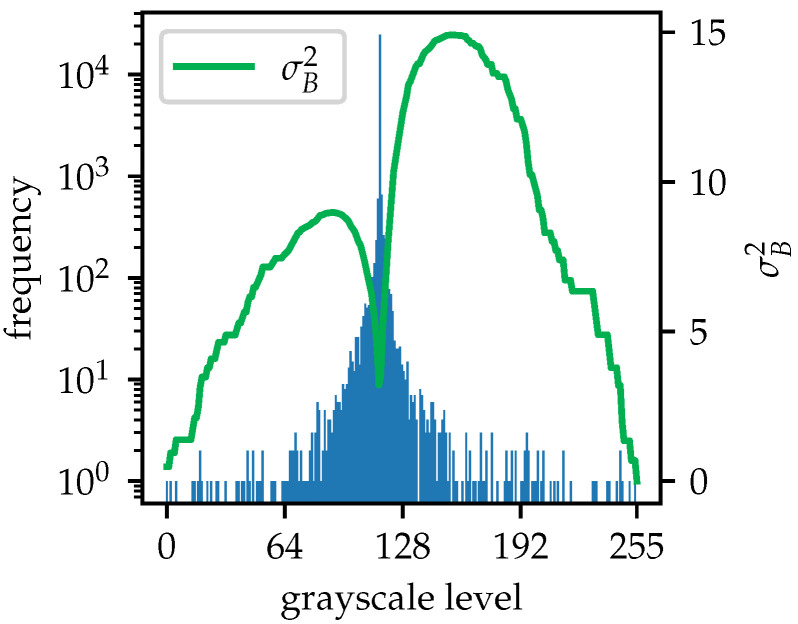
Grayscale histogram (blue bars) and interclass variance $\sigma_{B}^{2}$ curve (green line) of the enhanced image.

**Figure 9:**
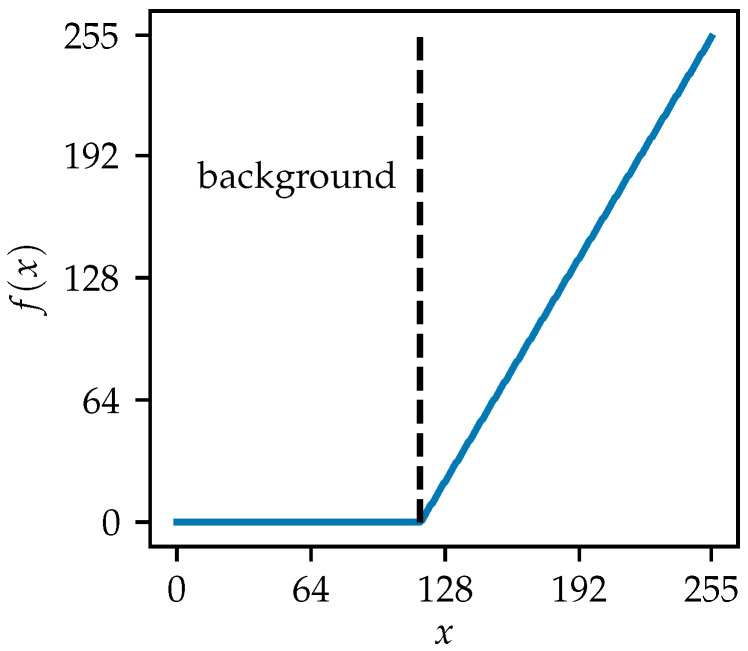
Mapping curve of the shift-and-scaling method f(·), where the dashed line indicates the grayscale level of the background.

**Figure 10:**
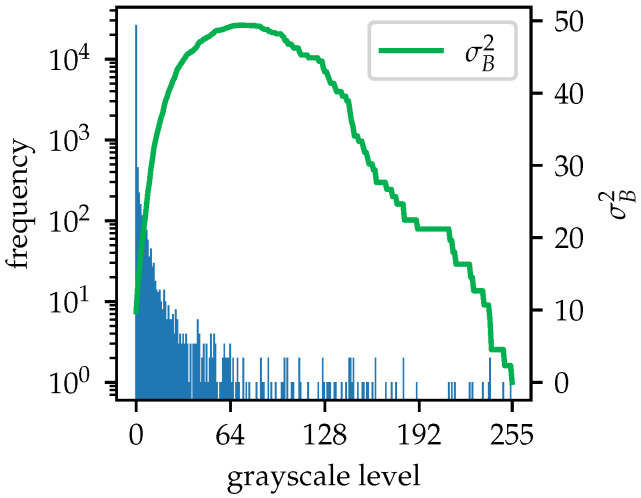
Grayscale histogram (blue bars) and the interclass variance $\sigma_{B}^{2}$ curve (green line) of the image after the shift and scaling process.

In the equations above, p(i) denotes the probability of the grayscale level *i*. Once $t^{*}$ is determined, the image can be thresholded by

(12) $$J\left(x,y\right)=\left\{\begin{array}{cc} I(x,y),\; & I\left(x,y\right)\geq t^{*},\\ 0,\; & I\left(x,y\right)<t^{*}, \end{array}\right.$$

where *I* and *J* are the original and thresholded images, respectively, and where (x,y) refers to the pixel at these coordinates. Global thresholding typically sets pixels with values greater or equal to $t^{*}$ to *L*, and to zero otherwise. However, since a subsequent local thresholding step is required, pixels that have been set to *L* retain their original values. The global thresholding is only designed to filter out pixels with values less than the threshold, removing useless information. Before thresholding, the image is converted to the Uint8 format, with pixel values ranging from 0 to 255. The grayscale histogram and the corresponding $\sigma_{B}^{2}$ curve plotted in Figure 8 characterize the image after applying Otsu’s method to Figure 3b. The $\sigma_{B}^{2}$ curve has two local maxima and a local minimum after a sharp drop at a certain grayscale level, which coincides with the background of the image. This histogram highlights uncertainty and a lack of confidence in determining a single threshold, as well as a narrow range of grayscale levels between the background and the maximum intensity. To address this issue, a background shift and grayscale scaling method is introduced. In the original B-scan, the pixels that carry significant information for hyperbola recognition and depth parameter determination have positive values. Other pixels primarily represent the negative parts of pulse signals or the near-zero background. In the grayscale-converted image, pixels with grayscale levels lower than the background are considered inessential. This method reassigns a grayscale level of 0 to the background and inessential pixels, while preserving the maximum value of 255. The grayscale range from the original background to the maximum is linearly scaled for all other pixels. The mapping curve and the histogram of the processed image are shown in Figure 9 and Figure 10, respectively. After applying the shift and scaling, grayscale levels greater than the background are spread more widely, increasing the differences among useful pixels. This enhances the precision of the binarization thresholding process.

**Figure 11:**
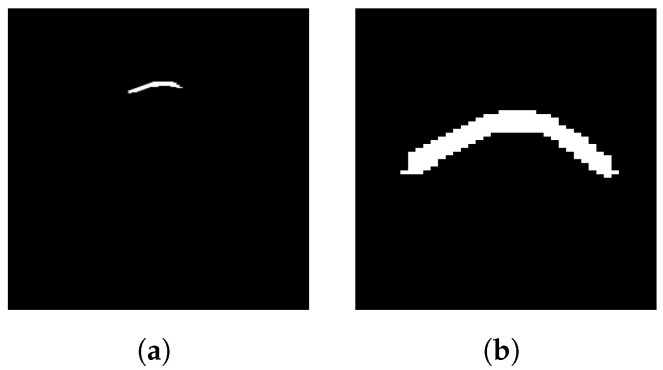
Results of thresholding. (**a**) Image thresholded by Equation (12). (**b**) Result of applying DLTS to (**a**) with a window size of 5 and k=0.5. The incomplete hyperbola in (**a**) has been well completed, providing preliminary validation of the effectiveness of DLTS.

The thresholded image, obtained by applying the threshold from Equation (12), which is calculated using Otsu’s method, is shown in Figure 11a. In this image, all ground reflection strips and background signals are completely removed, but a significant portion of the right half of the main hyperbola is missing. As a result, the main hyperbola is no longer intact, making it nearly impossible to determine its parameters. This outcome suggests that the selected threshold is larger than needed.

### 3.3. Local Thresholding and Hyperbola Segmentation

To reattach the tails to the head of the hyperbola, local features of the hyperbola are investigated by applying smaller, specified thresholds to less intense pixels. Since the proximity properties of each pixel must be analyzed, this process tends to be computationally intensive. As a result, the mathematical basis of most existing local thresholding methods is straightforward. The local mean and contrast are often suitable metrics for this purpose. Hasan introduced an approach that considers these factors:

(13) $$t\left(x,y\right)=k\left(\mu +\frac{I_{\max}-I_{\min}}{M}\right),$$

where *t* denotes the local threshold; *I* denotes the grayscale level; μ is the local mean; *M* is the global maximum of the grayscale level; and *k* is a control factor [28]. Once the local thresholds are determined, the thresholding operation is performed as follows:

(14) $$J\left(x,y\right)=\left\{\begin{array}{cc} 255,\; & I(x,y)\geq t(x,y),\\ 0,\; & I(x,y)<t(x,y). \end{array}\right.$$

Applying a local threshold to every pixel in the image can be computationally expensive when efficiency is a priority. Since the head of the hyperbola has been successfully revealed, applying the local method only in the proximity of the head is more efficient. To address this, an algorithm named dilation-based local thresholding and segmentation (DLTS) is introduced. DLTS searches the neighboring outer pixels along the edge of the shape to determine whether they should be thresholded in (set to 255) or out (set to 0).

Dilation is a morphological process [29]. Given that foreground A and kernel B are two sets in the two-dimensional integer space $Z^{2}$, which represents a space of two-dimensional images formed by pixels, the dilation of A by B, denoted as A⊕B, is defined as

(15) $$A⊕B=\left\{z\;|\;{\left(\hat{B}\right)}_{z}\cap A\neq ⌀\right\},$$

where $\hat{\cdot }$ denotes the reflection of a set, meaning that the reflected set and the original set are centrally symmetric about their origin; ${\left(\cdot \right)}_{z}$ denotes the translation of a set, where the translated set has been displaced by $z=(z_{1},z_{2})$ on the plane; ·∩· refers to the intersection of sets; and ⌀ represents the empty set. The dilation tries to repeatedly add the kernel to the foreground by traversing every pixel of the foreground, causing the foreground to grow. The DLTS applies dilation to the initial shape of the hyperbola head and performs local thresholding using Equations (13) and (14). In general, the kernel used for dilation is of odd × odd size, such as 3×3. A 3×3 kernel can expand a solid shape like a hyperbola outward by one pixel in all directions. The pixels that remain are united with the initial shape and form a new shape through subprocesses of checking each pixel in a local window to determine whether it should be thresholded in or out. Once the dilation reaches the boundary of the target hyperbola, pixel growth in that direction is stopped. When growth is no longer possible in all directions for each pixel, DLTS completes its process, the hyperbola is fully reconstructed, and both local thresholding and hyperbola segmentation are accomplished simultaneously. The DLTS process is expressed algorithmically in Algorithm 1.
**Algorithm 1** Dilation-based local thresholding and segmentation (DLTS).  1:Let H be the set of all pixels that form the thresholded hyperbolic shape, for example, the white region in Figure 11a, and E be a 3×3 set. Let N denote the set of neglected pixels, marking marginal areas to be skipped.  2:Let the edge of H be L. Update the edge with L=L−N. Let α=0 be the flag argument. Given the rightmost and bottommost pixel of H as $h_{0}$, and the central pixel of E as $e_{0}$, superimpose $h_{0}$ with $e_{0}$. Define *z* as the current position of the central pixel of E, which will be dynamically updated. Let $z_{0}=z$, and initialize $z^{\prime }=\left(-1,-1\right)$.  3:**if** $z_{0}\in N$**then**  4:   Skip to step 6.  5:**end if**  6:**Dilation:** Let $D={\left(E\right)}_{z}-{\left(E\right)}_{z^{\prime }}-H$.  7:**Local thresholding:** Apply local thresholding to every pixel in D using Equations (13) and (14).  8:**if** any pixel p∈D is thresholded in **then**  9:   Update H=H∪{p} and set α=1.10:**else**11:   Update N=N∪{z}.12:**end if**13:Move the center of E counterclockwise along L to the nearest unvisited position after setting $z^{\prime }=z$. Return to step 3 and repeat until $z=z_{0}$.14:**if** α=1 **then**15:   Return to step 2.16:**else**17:   The algorithm ends, and H represents the completed hyperbola.18:**end if**

However, based on the characteristics of most radar systems and the typical appearance of pipeline hyperbolas in B-scans, although hyperbolas may sometimes appear deformed or damaged, they almost never contain holes. One common application of dilation is to reduce or fill holes within the foreground, but this is not required for the task. Instead, DLTS only needs to progressively expand the foreground boundary until a well-formed hyperbola is reconstructed. Therefore, in each iteration, DLTS traverses only the boundary pixels of the hyperbola, which significantly reduces the computational burden. On the other hand, local thresholding is a widely used image processing technique that is more effective than global thresholding in capturing local information, but it typically comes with an increase in computational cost. Many local thresholding methods focus on statistical measurements such as the local maximum, mean, or variance, or combinations of these through operations. However, the exact local region, usually homogeneously windowed, is often left unspecified. This is natural, as in images that describe objective things, contextual information tends to be more important than precise spatial relationships. In contrast, hyperbolas are shape-sensitive targets, and an effective segmentation algorithm must take into account the relative positions of the pixels being absorbed through dilation. The inherent logic of dilation, as a morphological operation, restricts the scope of local thresholding to a small neighborhood immediately outside the boundary of the foreground. Therefore, combining local thresholding with dilation better aligns with the specific requirements of hyperbola segmentation tasks.

In practice, the determination of L is achieved using the border-following algorithm first introduced by Suzuki and Abe [30]. In Step 4, the previous instance $E_{z^{\prime }}$ of $E_{z}$ from the last iteration is subtracted from $E_{z}$ to avoid repeated calculations in Step 5. The value of $z^{\prime }$ is initialized as (−1,−1) to ensure that the subtraction of $E_{z^{\prime }}$ has no effect during the initial iteration.

After the image is enhanced by DGFE, globally thresholded by Otsu’s method, and adjusted through shifting and scaling, DLTS converges as long as the window size is smaller than the space between the main hyperbola and its primary multiple, with the background values remaining near zero throughout. The result of DLTS is shown in Figure 11b with a window size of 5 and k=0.5. The damaged hyperbola is clearly sufficient for shape recognition and parameter estimation, and no interference, such as ground reflection strips or multiples, is present.

## 4. Experiment

### 4.1. Results of Different B-Scan Implementations

**Figure 12:**
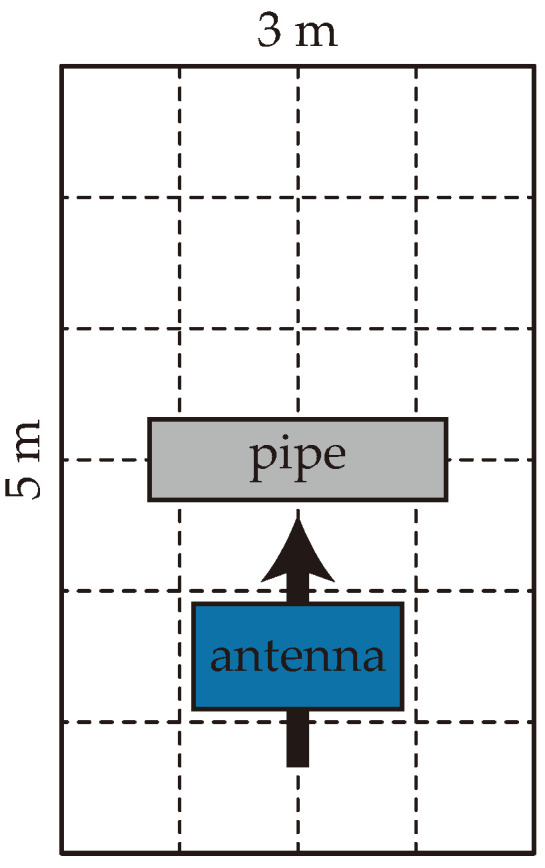
Scheme of the experimental field where Dataset 1 was collected. The 5.0 m ×3.0 m box was divided into a grid and the GPR moved perpendicular to the axis of the pipe.

To validate the proposed model’s algorithms, a number of B-scans were randomly selected from datasets collected under different operating conditions. These two datasets are hereafter referred to as Dataset 1 and Dataset 2. Dataset 1 was obtained from a field experiment conducted in Changji, Xinjiang Province, China. The soil in the area is a mixture of saline–alkali soil and sand, which creates suboptimal detection conditions. The experimental area was a rectangle of size 5.0 m (length)×3.0 m (width). The box was divided into a 6×4 grid, as shown in Figure 12, with every grid of size 0.75 m ×0.83 m. The GPR device was an ImpulseRadar CO1760 with a 600 MHz antenna (ImpulseRadar, Malå, Sweden). The pipe was buried underground with its cylindrical axis perpendicular to the long side of the experimental rectangle so that the pipe hyperbolas were captured by moving the GPR device perpendicular to the pipe axis. In the experiment, ductile iron pipes with outer diameters of 10, 15, and 20 cm were buried underground at a depth of 10 cm. The soil in the area is a mixture of saline–alkali soil and sand, which creates suboptimal detection conditions. Owing to the small radii of the pipes, the horizontal expansion of the hyperbolas was limited. As mentioned above, the model was designed to address these challenges.

**Figure 13:**
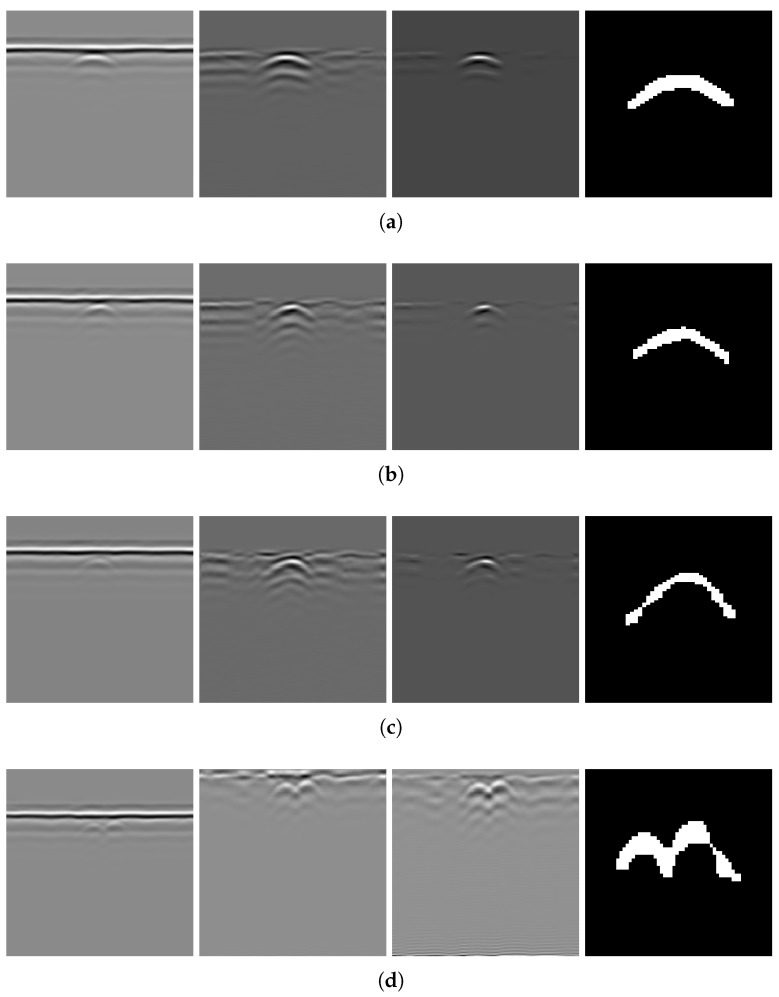
Results of the workflow applied to different B-scans from Dataset 1. In each row, the four subfigures from left to right are the original B-scan and the outcomes of GRRA, DGFE, and DLTS. (**a**) Pipe with a diameter of 20 cm. (**b**) Pipe with a diameter of 15 cm. (**c**) Pipe with a diameter of 10 cm. (**d**) Two pipes buried in parallel, with diameters of 15 cm and 20 cm. Pipes in (**a**–**c**) are buried at a depth of 10 cm, and pipes in (**d**) are buried at depths of 10 cm (one with 15 cm diameter) and 13 cm (one with 20 cm diameter).

**Figure 14:**
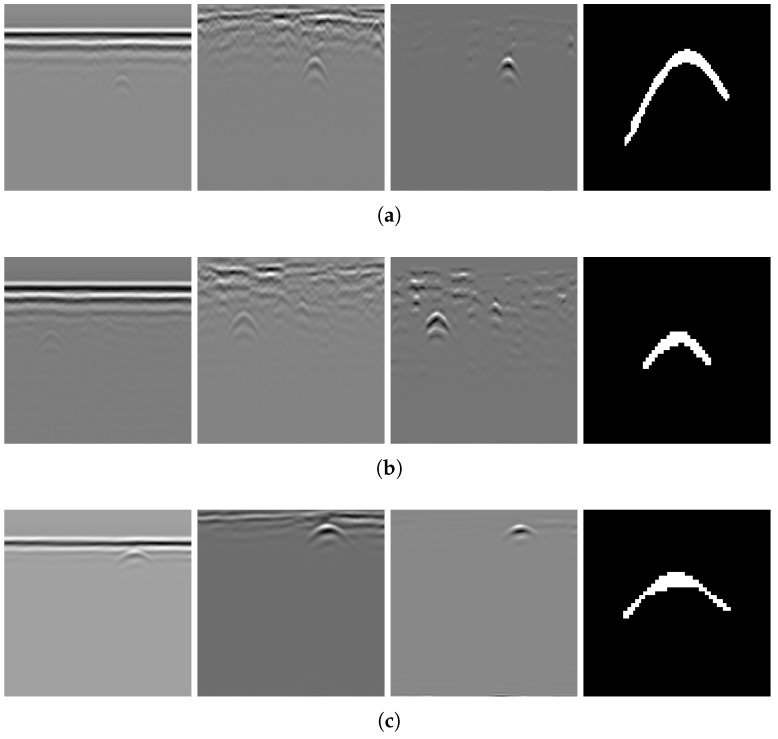
Results of the workflow applied to different B-scans from Dataset 2. (**a**,**b**) were acquired using a 600 MHz antenna, while (**c**) was acquired using a 170 MHz antenna.

Dataset 2 was collected from a residential area located in a relatively busy urban district of a city using the same GPR device with a dual-frequency antenna of 170 MHz and 600 MHz. For the ease of reproduction, the operation configurations of the GPR device when collecting Datasets 1 and 2 are listed in Table A1 and Table A2, respectively, in Appendix A. This area consists primarily of residential zones and functional zones, with multilayer asphalt pavement and heterogeneous soil conditions in the backfill layer. A variety of underground pipelines, including those for providing water supply, heating, drainage, and electricity, are buried beneath the road surface. These pipelines differ in material and burial depth, and in certain regions, their spatial arrangements are geometrically complex. Additionally, the site is subject to substantial electromagnetic interference from multiple sources. Compared to the operating environment in Dataset 1, the conditions in Dataset 2 are significantly more complex. It can be observed in Figure 13 and Figure 14 that the B-scan from Dataset 2 contains numerous additional reflections below the ground, which are caused by the complex subsurface structure of the pavement. Moreover, small, densely arranged hyperbola-like clutter is visible, indicating the presence of electromagnetic interference and structural heterogeneity.

To quantitatively assess the differences between Datasets 1 and 2, the JSUM signal-to-clutter ratio (SCR) metric is adopted to evaluate the relative prominence of target features with respect to background noise because the target is more easily interfered with by non-target reflections than general noises [31]

(16) $$J_{\mathrm{sum}}=\frac{1}{N}\sum_{i=1}^{N}{\left[\frac{\left(t_{i}-\mu_{i}\right)}{\sigma_{\mathrm{bkg}}}\right]}^{2}.$$

Within the preselected background region, each A-scan is associated with a corresponding SCR metric. According to common practice, all SCR values within this region are averaged to serve as a measure of the target’s prominence relative to the background or clutter. In Equation (16), *N* is the number of time samples of the A-scan; $t_{i}$ and $\mu_{i}$ are the data value and the average value of background at *i*th time bin, respectively; and $\sigma_{\mathrm{bkg}}$ is the average standard deviation of the background A-scans. The SCR is a quantitative measure specifically designed for assessing the effectiveness of B-scan preprocessing, and it can also assess the extent of distinction of the target from the background. A larger SCR value indicates a more prominent target and thus better performance of the background removal method. In the practical calculation, all A-scans in the B-scan that do not contain any hyperbolas are manually selected as the background A-scans used in the computation. The typical SCRs of the two datasets are listed in Table 1, along with the burial depths of the pipes. As shown in Table 1, the signals in Dataset 2 are typically less distinct from the background than those in Dataset 1, which corresponds to the more complex acquisition surroundings in Dataset 2. And all pipes in the residential area where Dataset 2 was collected are buried much deeper than those corresponding to Dataset 1.

Applying the algorithm to B-scans from two different datasets can provide evidence of its generalizability across varying conditions. The results of several B-scans from Dataset 1 are shown in Figure 13. From subfigures (a) to (c), the figure displays B-scans of pipes with diameters of 20, 15, and 10 cm and a burial depth of 10 cm, while subfigure (d) displays pipes with diameters of 15 and 20 cm, buried in parallel, at depths of 10 and 13 cm, respectively. From left to right, the four subfigures sequentially show the original B-scan and the outputs of GRRA, DGFE, and DLTS. In GRRA, c=1 and γ=1.3. For DLTS, the window size was set to 5, and k=0.5. Although fixed parameter values are used here, how variations in these parameters affect the performance and effectiveness of the algorithms will be discussed later. At a burial depth of 10 cm, the pipes are fairly distinguishable from the background by the human eye. However, at a depth of 15 cm, the images are harder to discern because of the small radius of the pipe, poor medium conditions, and limitations in the principles of electromagnetic wave propagation. This highlights the need for the algorithm to adapt to challenging conditions. The results indicate that, for pipes buried at shallower depths, the model performs reliably. Additionally, it successfully addresses scenarios where there is minimal contrast between the hyperbola and the background. When the workflow is applied to B-scans containing two overlapping hyperbolas, the situation becomes somewhat different. Under the condition that the overall reflection intensity of the hyperbolas is rather low, the enhancement effect of DGFE is less pronounced than that observed for isolated hyperbolas. This is because, according to the superposition principle and the image, the signal amplitude at the overlapping region is higher than that of the surrounding areas, producing a sudden high-intensity peak along the hyperbola tails. Such a peak weakens the algorithm’s distinction between foreground and background features. Nevertheless, the final output still largely preserves the shape of both hyperbolas, although their spread is less uniform than in the case of non-overlapping ones. In addition, the hyperbolas are overall wider, which can be attributed to the global thresholding retaining more signals when the intensity difference between foreground and background is relatively small. This further demonstrates the necessity of considering reflection intensity during the preprocessing stage. The results of applying the workflow to B-scans from Dataset 2 further support the point, demonstrating its applicability in cases where the intensity contrast between hyperbolas and the background is low. Figure 14 presents the results obtained using the algorithms on B-scans acquired with two different antenna frequencies, with parameter values kept unchanged. It can be observed that the hyperbolas were effectively extracted in all cases.

As shown in Figure 13 and Figure 14, GRRA, DGFE, and DLTS each fulfill their intended design purposes, and the output images exhibit clear characteristics. GRRA applies a Fourier domain filter with a vertical DC damper, effectively removing horizontal features, such as ground reflection strips. However, this comes at the cost of enhancing other vertically periodic features, especially the multiple-like curved strips beneath the target hyperbola. DGFE is designed to mitigate the negative effects of GRRA and further enhance the intensity of the target hyperbola. As a result, other components in the images become darker as the target hyperbola becomes brighter. This effectiveness is also clearly demonstrated in the more complex subsurface conditions of Dataset 2, where DGFE effectively suppresses clutter intensity. Despite this, the tails of the hyperbola remain insufficiently bright and can be easily missed by a global threshold. This highlights the importance of DLTS, which completes the hyperbola by preserving its shape. The proposed model demonstrates its effectiveness in extracting the target hyperbola from the raw B-scan while maintaining its shape. Additionally, to assess the speed of the proposed model to meet the demand of edge deployment, 80 B-scans under the same experimental conditions from Dataset 1 and 30 B-scans from Dataset 2 were used as inputs. The runtime statistics, measured on an Intel^®^ Core™ i7-10700 CPU, are presented in Table 2, where the image size of Dataset 1 is 300×120 (height × width), and that of Dataset 2 is 240×257. The results indicate that the model’s algorithms are both swift and stable.

As for algorithm complexity analysis, since the pipeline consists of multiple modules, it is conducted sequentially. Let *S* (sample) and *T* (trace) denote the height and width of the B-scan image, respectively. (1) GRRA includes a Fourier domain filter, where the two-dimensional fast Fourier transform and its inverse operation both have a complexity on the order of O(S·T·log(S·T)), with an fftshiftfunction on the order of O(S·T). Fourier domain filtering features the multiplication of the Fourier spectrum and the transfer matrix, whose complexity is on the order of O(S·T). Similarly, Equation (5) is also on O(S·T). Therefore, the computational complexity of GRRA is on the order of O(S·T·log(S·T)). (2) DGFE has two layers of loops. The outer loop moves a two-dimensional window with the size of W×W for S·T times, and the inner loop conducts the operations between matrices with the same size of W×W. Therefore, the computational complexity of DGFE is on the order of $O(S\cdot T\cdot W^{2})$. (3) The thresholding module includes global thresholding and DLTS. The global thresholding is also on O(S·T). As for DLTS, its initialization traverses the foreground and builds statistic matrices such as means, minimum and maximum values, and contrasts, with complexity on the order of O(S·T). Other contributing computations are the outer and inner loops; the step with the heaviest computation is determining the edge at the start of each outer loop, on the order of O(P), where *P* is the number of pixels contained in the foreground. Therefore, the complexity of thresholding is on the order of O(S·T). Since the modules are processed sequentially and without nesting, the computational complexity of our proposed model is on the order of $O(S\cdot T\cdot \max\left(W^{2},\log\left(S\cdot T\right)\right))$. The steps with the heaviest computation are filtering in GRRA and image enhancement in DGFE, both containing matrix multiplications and accumulations. As suggested by the complexity analysis and Table 2, the computational cost is quite acceptable.

Notably, the extracted hyperbola in Figure 13c appears thin and slightly distorted. On the basis of the equation of a hyperbola and the continuous form of Equation (1), where x=bcosht, y=asinht, the two-dimensional Fourier transform $\hat{H}$ of the hyperbola is

(17) $$\begin{array}{cc} \hat{H}\left(\hat{x},\hat{y}\right) & =\int_{-\infty }^{\infty }\exp\left[-j2\pi (\hat{x}b\cosh t+\hat{y}a\cosh t)\right]\;dt \end{array}$$

(18) $$\begin{array}{cc} \; & =2K_{0}\left(2\pi \sqrt{{\left(b\hat{x}\right)}^{2}+{\left(a\hat{y}\right)}^{2}}\right), \end{array}$$

where $K_{0}\left(x\right)$ denotes the modified Bessel function of the second kind at ν=0 [32]. In the neighborhood of x=0, $K_{0}\left(x\right)>0$, and it quickly decays to 0 as |x| increases. The frequency-domain filter used by GRRA passes through the proximity of the origin of the Fourier plane, setting it to 0, where key features of the hyperbola reside. As a result, the shape of the hyperbola is affected, and the less intense the hyperbola is, the more pronounced the influence becomes.

To verify the effectiveness of the algorithm, a hyperbola needs to be fitted from the binary image output by the entire algorithm flow. The extracted hyperbola parameters are then compared with the experimental conditions via Equation (19).

(19) $$\begin{array}{c} {\left(\frac{\tau +2R/v}{\tau_{0}+2R/v}\right)}^{2}-{\left(\frac{x-x_{0}}{v\tau_{0}/2+R}\right)}^{2}=1, \end{array}$$

where τ and *x* refer to the two axes of the image, vertically and horizontally, respectively; *R* is the outer radius of the pipe; *v* is the electromagnetic wave speed in the medium, which equals $c/\sqrt{\varepsilon_{r}}$, where *c* is the speed of light; $\varepsilon_{r}$ is the dielectric constant of the medium; and $\tau_{0}$ is the round-trip time of the electromagnetic wave, which equals 2h/v, where *h* is the burial depth of the pipe. Among these parameters, the burial depth *h* and the outer radius of the pipe *R* are important and can be compared with the experimental scenarios. The round-trip time $\tau_{0}$ is directly deduced from the position of the topmost pixel of the hyperbola, whereas the outer radius *R* is deduced from Equation (19). The selected real-world dataset from Dataset 1 consists of three scenarios, where pipes with outer diameters of 20, 15, and 10 cm are buried at a depth of 10 cm. Each scenario includes 20 different B-scans, which vary owing to perturbations in the positions and angles of survey lines. The fitting method adopts the least-squares method from [18], as shown in Equation (20), which is deduced from Equation (19). This process requires fitting a set of four parameters $\left(a,b,R,x_{0}\right)$ from the set of hyperbola pixels in the output binary image.

(20) $$\tau =-\frac{2R}{v}+a\sqrt{1+\frac{{\left(x-x_{0}\right)}^{2}}{b^{2}}},$$

where $a=\tau_{0}+2R/v$, $b=v\tau_{0}/2+R$, and v=2b/a.

The algorithm is evaluated with k=0.7 and a window size of 5, and the validation results are presented in Table 3. In the table, $R_{\mathrm{actual}}$ (unit: cm) refers to the actual outer radius of the pipe, ${\bar{R}}_{\mathrm{est}}$ (unit: cm) refers to the mean estimated value of the outer radius with the algorithm, and ${\bar{\mathrm{error}}}_{R}$ refers to the mean relative error of estimation; the symbols are similarly applied to *h*, which is the burial depth. Table 3 displays the range of pipe burial depths and outer diameters derived from the fitted hyperbola parameters in successful trials, along with the average relative error. Additionally, among the 20 B-scan images for the pipe with an outer diameter of 10 cm, 3 B-scans failed to produce a complete hyperbola, resulting in truncated tail sections instead. The algorithm performs reasonably well when applied to pipes with outer diameters of 20 and 15 cm, as it can accurately estimate the burial depth and outer radius. However, when it is applied to 10 cm pipes, the performance has a minor decline due to the faint hyperbolic signatures in the raw B-scan images. Furthermore, the estimated burial depth tends to be greater than the actual depth and the outer radii are smaller, which may be attributed to the following factors: (a) In the experimental setup, the process of excavation, pipe placement, and backfilling may cause soil compaction and pipe settlement, leading to an actual burial depth greater than the designed depth. (b) The initial position determined by the filter via Equation (5) may deviate from the actual initial position of the first ground reflection strip, introducing estimation bias.

**Figure 15:**
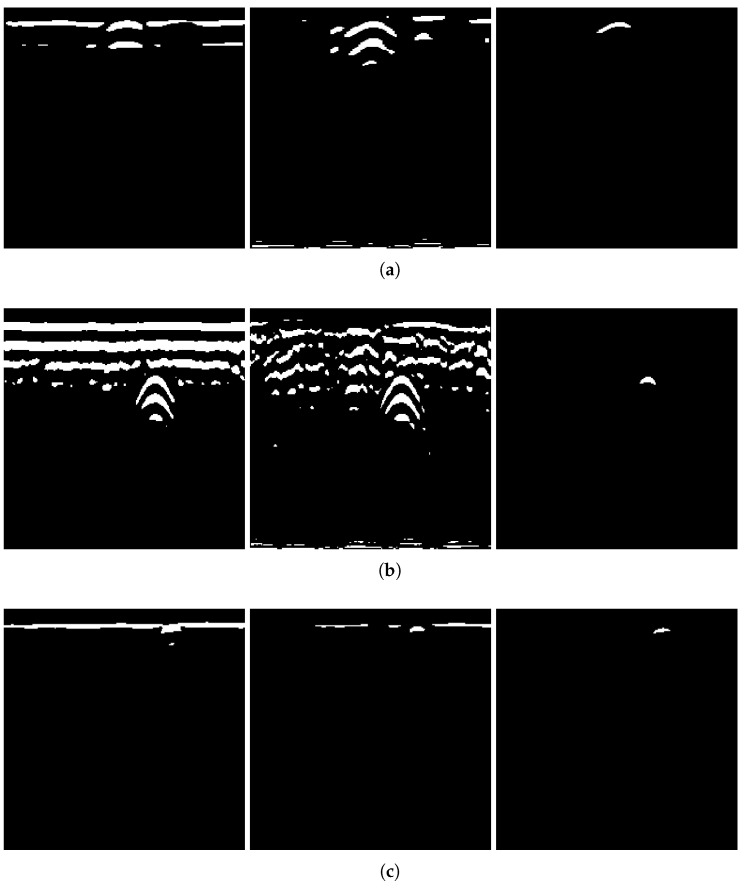
Results of the different algorithmic variants of the ablation study of B-scans: (**a**) in Figure 13a; (**b**) in Figure 14a; (**c**) in Figure 14c. In each subfigure, the results from left to right correspond to variants (**a**), (**b**), and (**c**), respectively.

To verify the necessity of the algorithm components—GRRA, DGFE, and the DLTS-based thresholding and segmentation method—in the proposed model, an ablation study was conducted. Specifically, GRRA, DGFE, and DLTS were removed one at a time to form three alternative algorithmic variants: (a) DGFE followed by DLTS-based thresholding and segmentation; (b) GRRA followed by DLTS-based thresholding and segmentation; (c) GRRA, then DGFE, and finally thresholding without DLTS. These three variants were applied to B-scans from both Dataset 1 and Dataset 2, and the corresponding results are shown in Figure 15. As shown in the figure, the outputs of the three algorithmic variants, each lacking a specific step, are affected to varying degrees by the absence of that step. The extent of the impact depends on the characteristics of the data acquisition environment in each dataset. Variant (a) does not specifically handle the strong ground reflection strips. Since the intensity of the ground reflection is significantly higher than that of the hyperbola, both may be retained during thresholding due to their similar intensities even after gain. In some cases, the hyperbola may even be discarded. Variant (b) lacks dedicated suppression of non-hyperbola interference. This issue is particularly evident in Dataset 2, where the presence of significant clutter and the absence of image enhancement cause the hyperbolas to exhibit intensities comparable to the background. As a result, both may be preserved during thresholding, and in some cases, the hyperbolas may be lost. Variant (c) has already been discussed earlier: the extracted hyperbola suffers from varying degrees of incompleteness, which highlights the necessity of the DLTS step for recovering missing hyperbola components. The ablation study provides evidence for the rationality and necessity of each step in the proposed model. The effectiveness of the overall algorithm relies on the combined contribution of all components, and none of them can be omitted without compromising performance.

### 4.2. Comparison with Other Algorithms

In terms of horizontal comparison with existing preprocessing methods, the overall algorithmic pipeline proposed in this paper performs functions analogous to conventional background removal through the GRRA and DGFE modules. Subsequently, the DLTS-based image thresholding module achieves hyperbola segmentation. In the following subsections, the performance of the proposed method is compared with that of recent approaches developed in the past few years in two aspects: (a) ground reflection and background removal and (b) end-to-end performance.

A commonly used method for B-scan image background removal is derived from the running average filter, also known as the moving average filter, which is a classical and widely used technique in the field of signal processing. It computes the average value $y_{i}$ of the neighborhood around a given point $x_{i}$ in a signal sequence as follows:

(21) $$y_{i}=\frac{1}{M_{1}+M_{2}+1}\sum_{k=-M_{1}}^{M_{2}}x_{i-k},$$

where $M_{1}$ and $M_{2}$ define how many data points before and after the current index *i* are included in the average; thus, the denominator $M_{1}+M_{2}+1$ is the total number of points being averaged [33]. Typically, the running average filter is employed for signal smoothing. However, by subtracting the computed average $y_{i}$ from $x_{i}$, background removal can be achieved. This approach is generally referred to as mean subtraction.

Mean subtraction can be performed one-dimensionally, row by row, as implemented in [17,18,19], according to the following expression:

(22) $$E_{x,y}=E_{x,y}-\frac{\sum_{x=1}^{m}E_{x,y}}{m},$$

where $E_{x,y}$ represents the value at (x,y), and *m* is the number of signal points; in this case, $m=M_{1}+M_{2}+1$ in Equation (21). Alternatively, it can be applied two-dimensionally within a moving local window. In addition, ref. [18] further employed a filter known as the median filter. By sequentially applying these two filters, it is possible to suppress antenna ringing, eliminate ground reflection strips, and mitigate radiofrequency interference. In the following, the performance of DGFE followed by GRRA will be quantitatively compared with that of the combined approach of the mean subtraction method described by Equation (22) and median filtering used by [18]. Next, the above three methods for background removal will be applied to the B-scans from Dataset 1 (group A) and Dataset 2 used in the previous part, with the latter including B-scans acquired using both 600 MHz (group B) and 170 MHz (group C) antennas. The target region is defined as a box that extends laterally by one hyperbola width, determined by the result of DLTS, on each side of the target, and vertically from the topmost line of the ground reflection down to two hyperbola heights beneath the target. This ensures that the hyperbola lines are within a reasonable surrounding background region and that the SCR value if not overly diluted by a large background area. In the following discussion, all reported SCR values refer to the average SCR within the selected background region, further averaged over all sampled B-scans.

**Figure 16:**
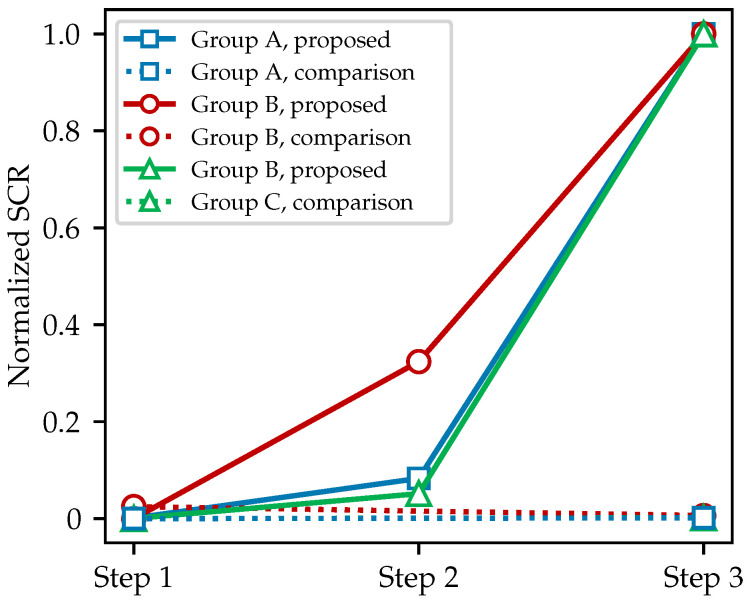
Comparison between the proposed workflow and a commonly used method regarding the performance of background removal. The normalized SCR serves as the metric.

Figure 16 shows line plots comparing the SCR values at each step of the background removal process, applied to three groups of B-scans. The proposed model uses a sequence of operations, namely, GRRA (step 1), gain introduced by Equation (5) (step 2), and DGFE (step 3), and is labeled proposed in the legend; the comparison method applies mean subtraction (step 1) followed by a median filter with a window size of 5 (step 3) and is labeled comparison in the legend. To facilitate a fair comparison of the two methods on the same B-scans, all SCR values in this figure were normalized within each group: the minimum value in each group was set to 0, and the maximum to 1. The clear separation between the solid and dashed lines in Figure 16 indicates that, according to the commonly used SCR metric, the background removal workflow proposed by this paper significantly outperforms the conventional approach. In fact, after intra-group normalization, the contribution of the median filter to SCR improvement becomes nearly imperceptible.

**Figure 17:**
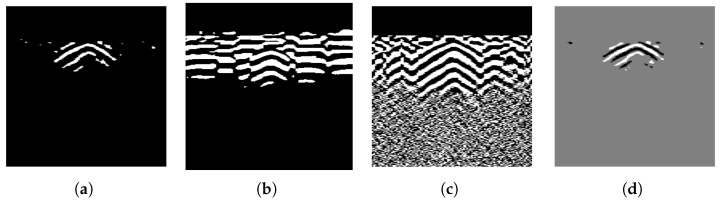
Results of different mono-level and bilevel global thresholding methods: (**a**) minimum cross-entropy method; (**b**) Dai’s method based on Canny edge detection and lobe ratio; (**c**) vertical gradient method; (**d**) multilevel Otsu’s method.

The integration of local thresholding into the thresholding and segmentation process is essential. Figure 17 shows thresholded images produced by several mono-level and multilevel methods, including the minimum cross-entropy method introduced by Li and Liu [34], adaptive thresholding based on Canny edge detection and lobe ratio in Dai’s model [19], the vertical gradient method used in Zhou’s GPR model [18], and the multilevel Otsu’s method [35]. The multilevel Otsu’s method divides the image into n+1 bins by *n* thresholds, with n=2 applied in Figure 17d. In this case, pixels with grayscale values greater than the higher threshold are set to white to indicate the probable target hyperbola, values smaller than the lower threshold are set to black, and values between the two thresholds are set to gray. All four methods are clearly unsuitable for B-scans from the dataset of this paper. The outputs of these methods simultaneously retain both the target hyperbola and multiples. Moreover, the number of multiples is often large, appearing chaotically around the target hyperbola, which significantly affects subsequent processing. The key issue lies in two aspects. First, these thresholding methods fail to effectively differentiate between the target hyperbola and its multiples in terms of intensity. In general, the target hyperbola has a higher intensity than its multiples do, but this distinction is not well captured or leveraged. Second, to ensure that the algorithm completes execution within hundreds of milliseconds to a few seconds, commonly used processing algorithms often rely on global thresholding, which is rougher than local thresholding and sacrifices fine-grained image processing to save computational resources. As a result, the difference between the hyperbola and other interference signals is ignored in further analyses.

In fact, retaining unexpected multiples during the thresholding process is a common phenomenon. When Dai et al. applied several clustering-based hyperbola recognition and segmentation algorithms, including the open-scan clustering algorithm (OSCA), the column connection clustering algorithm (C3), and the hyperbolic trend clustering algorithm (HTCA), to real-world B-scans, 1–2 multiples were often observed beneath the target hyperbola [19]. These multiples were retained along with the hyperbola due to their shared geometric characteristics, such as a downward opening and two extending tails. Although these multiples were eventually discarded on the basis of their positional relationships, they still consumed computational resources during the recognition process. Clustering-based algorithms are widely used, but they share a common limitation. These methods focus primarily on morphological features while almost neglecting signal intensity information. This is precisely the issue that DGFE and DLTS aim to address.

Next, an end-to-end comparison is presented between the proposed algorithms and the three recently introduced cluster-based methods mentioned above. The comparison focuses on two main aspects: (a) the performance differences, as reflected in the processed images, especially the capability to deal with multiple reflections; (b) the differences in runtime. As before, the B-scans used in this comparison are drawn from both Dataset 1 and Dataset 2. Before analyzing the results, it is necessary to clearly describe the toolchain used for each method. Method A follows the sequence (a) mean subtraction and median filter, (b) nonlinear time gain, (c) gradient-based thresholding, (d) opening and closing, and (e) OSCA, as originally used in [18]. However, the component (c) gradient-based thresholding was replaced with a mono-level Otsu’s method in our implementation, because as shown in Figure 17c, it does not handle the B-scans in our datasets effectively. Method B follows the sequence (a) mean subtraction, (b) mono-level Otsu’s method, (c) opening and closing, and (d) DCSE, as used in [9]. Method C follows the sequence (a) mean subtraction, (b) adaptive thresholding based on Canny edge detection and lobe ratio, and (c) HTCA, as used in [19], but the thresholding method was also replaced with a mono-level Otsu’s method.

**Figure 18:**
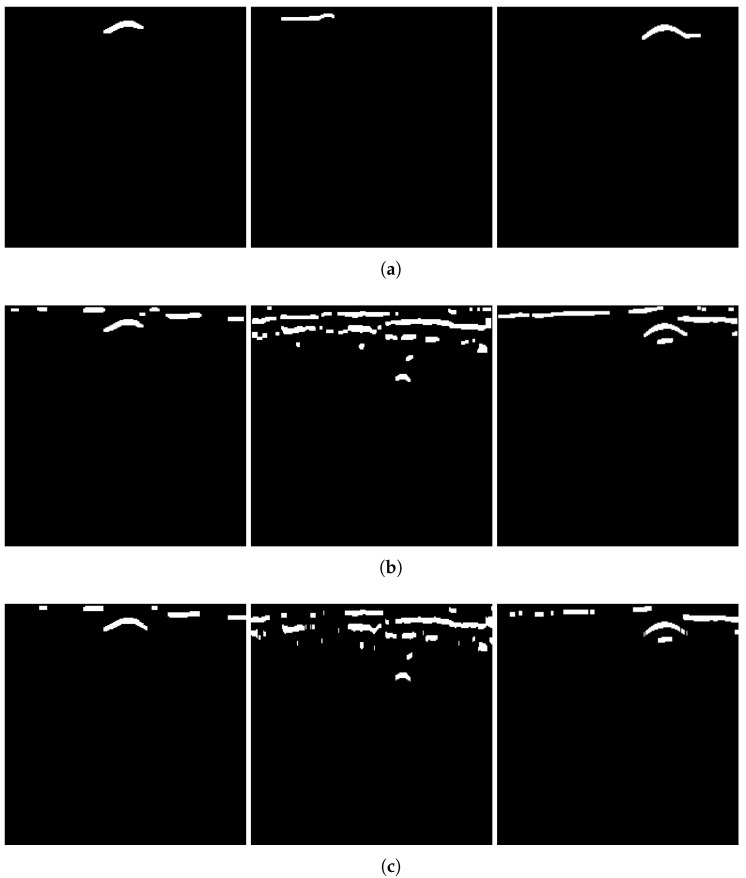
Results of applying the three methods to B-scans from the two datasets. It can be seen that, due to the lack of target-intensity-based image enhancement, these methods sometimes struggle to effectively suppress ground reflections and clutter. (**a**) Method A with OSCA; (**b**) Method B with DCSE; (**c**) Method C with HTCA.

Figure 18 presents the results of the three methods applied to three sample B-scans from Dataset 1 and Dataset 2. In many cases, these cluster-based methods are able to identify and extract the target hyperbolas, especially in scenarios with minimal ground reflection and clutter, where the hyperbolas are visually prominent, such as B-scans obtained using low-frequency antennas. However, since all three methods are morphological and cluster-based, they rely on geometric features such as the opening direction, the apex and tail of the hyperbola, and the spatial or intersecting relationships between hyperbola candidates. As a result, point clusters that happen to meet the predefined geometric criteria are often falsely included. These include, for example, horizontal strips with locally convex shapes, hyperbola-like clutter that was not removed, or artifacts caused by multiple reflections beneath real hyperbolas.

To quantitatively evaluate the characteristics of these four methods, they are applied to a group of samples containing 40 B-scans drawn from Dataset 2, which were collected by the 600 MHz antenna. This group of data contains stronger and more apparent clutter compared to Dataset 1 and those from Dataset 2 that were collected by the 170 MHz antenna. Three statistical indicators are computed: (1) whether the output preserves the shape of the hyperbola, expressed as a Boolean variable whose arithmetic sum is denoted as $B_{\mathrm{shape}}$, to evaluate whether useful information is retained; (2) whether the hyperbola is connected to unrelated clusters, expressed as a Boolean variable whose arithmetic sum is denoted as $B_{\mathrm{connected}}$; (3) the average number of isolated clusters other than the target hyperbola, denoted as $N_{\mathrm{cluster}}$, to measure the cleanness of the processing results. The results are shown in Table 4.

The results show that the four methods exhibit comparable abilities to preserve useful information and eliminate unintended noise, except that Method A performs slightly worse. However, both the proposed method and Method A achieve significantly higher cleanness in the output results. This quantitative validation is consistent with the preliminary conclusion drawn from the visual inspection of the outputs. The comparison highlights the necessity of DGFE, the target-intensity-based image enhancement in the proposed toolchain, as it effectively suppresses non-target information before morphological processing. However, the morphological methods themselves do not alter the shape of the targets, nor do they introduce or remove pixels unintentionally, which can sometimes occur with DGFE. As a result, these cluster-based methods tend to better preserve the original shape of the hyperbolas, which is an inherent advantage of cluster-based hyperbola extraction approaches. This observation highlights the sensitivity of one method to the specific characteristics of the dataset.

Moreover, methods like OSCA focus solely on the relative spatial relationships among point sets, making them less computationally intensive than DGFE, which requires analysis of global pixel intensity patterns. DCSE further reduces complexity by simplifying point sets into point segments row-wise in a two-dimensional image, resulting in shorter runtimes. Table 5 compares the average runtime of the four methods on both datasets. It can be observed that all of them are computationally efficient, on the order of tens to hundreds of milliseconds, making them suitable for deployment on edge devices. Of course, beyond analyzing the topological and morphological characteristics of pixel sets, the proposed method also considers their intensity relationships based on spatial positions. This introduces a substantial number of matrix multiplications and accumulations, but leads to better overall performance on the datasets. This result demonstrates that the trade-off we made is worthwhile. After all, the processing speed is still around 10 FPS on a relatively modest CPU. This enables the proposed method to achieve above-average processing quality while still meeting the requirements for edge deployment.

### 4.3. Parameter Analysis of DLTS

**Figure 19:**
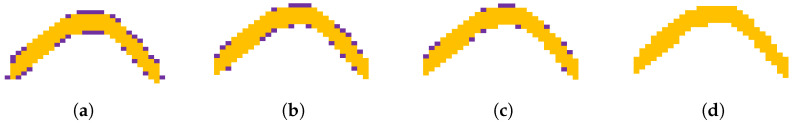
DLTS results for the example image (B-scan for a pipe with an outer diameter of 20 cm) at different values of *k* and a window size of 5: (**a**) k=0.5; (**b**) k=0.6; (**c**) k=0.7; (**d**) k=0.8. As *k* increases, the number of pixels in the target hyperbola decreases. Since the hyperbola in (**d**) contains the fewest pixels, in subfigures (**a**–**c**), the additional pixels included in the hyperbola compared with (**d**) are filled in purple, whereas the overlapping pixels among all figures are filled in yellow.

**Figure 20:**
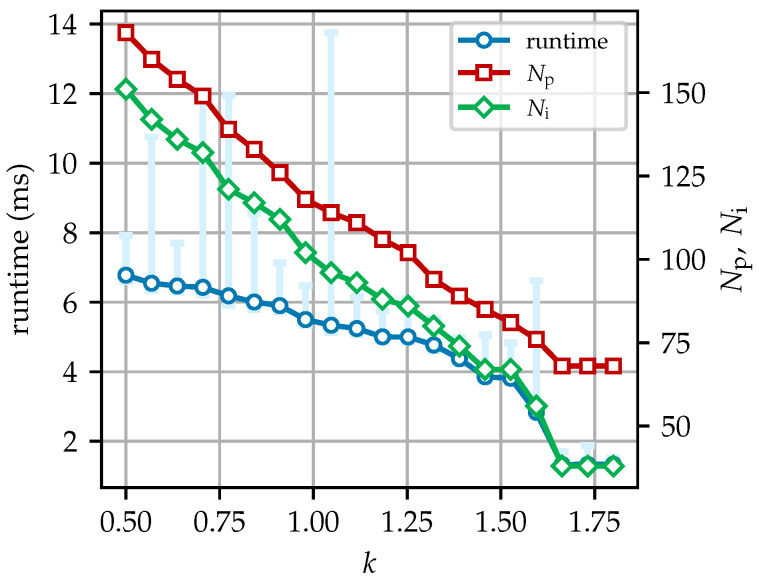
Changes in runtime, the number of pixels in the resulting hyperbola, $N_{p}$, and the number of iterations of sub-DLTS, $N_{i}$, as *k* varies.

The DLTS results from Figure 11a, which is the output of Otsu thresholding from a B-scan of a pipe with an outer diameter of 20 cm, using different values of *k* and the same window size of 5, are shown in Figure 19. As *k* increases, the local thresholds tend to rise, resulting in fewer pixels being set to 255, according to Equation (14), which makes the hyperbola appear thinner. As shown in Figure 20, the value of *k* affects not only the size of the hyperbola (i.e., the number of pixels in it, $N_{p}$) but also the runtime and the number of iterations, $N_{i}$, of the sub-DLTS procedure, as outlined in Steps 4 and 5 of the algorithm.

Figure 20 shows a general reduction in runtime, $N_{p}$, and $N_{i}$, with minor regional variations. In this figure, computation under the same conditions is conducted 1000 times to highlight runtime fluctuations, which are represented by the light-blue error bars. As is shown, the execution time of DLTS ranges from a few milliseconds to over ten milliseconds. As *k* increases, although the runtime can be reduced, with the average decreasing from approximately 7 milliseconds at k=0.5 to less than 4 milliseconds at k=1.4, the hyperbola shape may be compromised, as reflected in the reduction in $N_{p}$. Notably, when *k* reaches 1.5 or higher, there is a noticeable decrease in the runtime and $N_{i}$. However, once *k* reaches 1.6, $N_{i}$ and $N_{p}$ no longer decrease. According to Equation (13), the value of *k* controls the threshold for local thresholding. A larger *k* results in a higher threshold, leading to fewer retained pixels in the output hyperbola. Consequently, continuously increasing *k* may have adverse effects, potentially rendering the algorithm ineffective. Conversely, excessive iterations and fine computations are not always beneficial to the quality of the result, as shown in Figure 19a, where burrs appear on the hyperbola. In terms of runtime, regardless of the value of *k*, the average runtime always remains within a few milliseconds, and the maximum does not exceed 15 milliseconds. Therefore, although different *k* values cause significant variations in execution time, from a practical perspective, the impact of *k* on the runtime of the algorithm is negligible. Additionally, for many values of *k*, the average runtime is only slightly higher than the minimum runtime but significantly lower than the maximum. This right-skewed characteristic is a normal phenomenon, as only a small number of executions experience significantly longer runtimes due to occasional variations in the operating environment. The decreases in $N_{p}$ and $N_{i}$ halt when *k* becomes large enough, indicating that DLTS is no longer effective in completing the hyperbola. In practice, *k* should be fine-tuned on the basis of the results, starting from a recommended value of no larger than 1.25 to achieve an intact and uniform spread of the hyperbola.

Clearly, the larger the value of *k*, the stricter the resulting local threshold becomes. When *k* increases beyond a certain point, no new pixels will be absorbed into the foreground. Conversely, as *k* decreases, the threshold becomes more lenient. If it drops too low, even background pixels may be incorrectly included, which is highly detrimental and often leads to the failure of the algorithm. Therefore, a robustness and parameter range analysis was conducted to determine the effective range of *k* for the majority of B-scans. A total of 60 B-scans are sampled, 20 from Dataset 1, 20 from Dataset 2 acquired with a 600 MHz antenna, and 20 from Dataset 2 acquired with a 170 MHz antenna, to investigate the effective range of the parameter *k*. For each B-scan, the upper bound of *k* is determined based on the point at which no additional foreground pixels are absorbed. In contrast, the lower bound can only be identified through visual inspection, by observing the emergence of clearly non-hyperbola pixels that adversely affected the result. During the evaluation, the fineness of *k* adjustment is set to 0.01.

**Figure 21:**
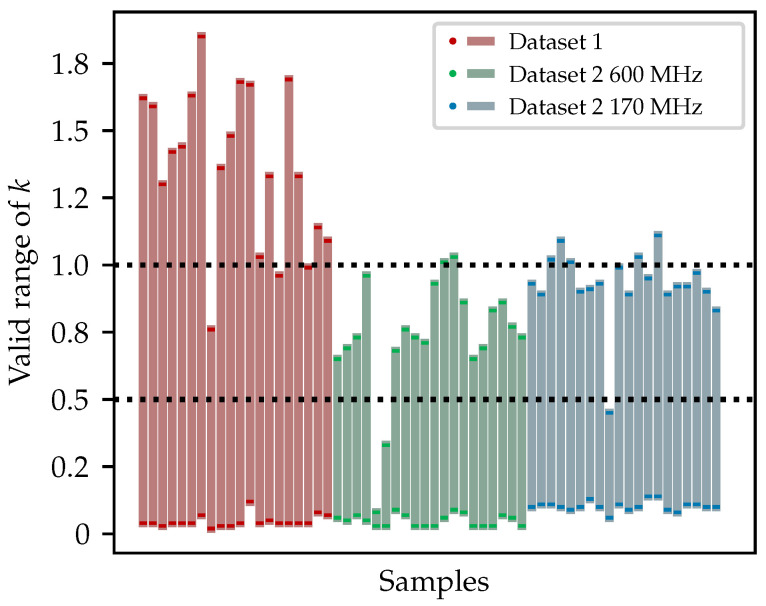
Valid *k* ranges of different B-scans from the two datasets. The two dotted horizontal lines indicate where k=0.5 and k=1.0.

Figure 21 shows the results of the robustness and parameter range analysis. To facilitate quantitative interpretation, two dotted horizontal reference lines at k=0.5 and k=1.0 are drawn. The results indicate that for B-scans from different datasets, *k* is effective over a reasonably wide range, with only a few instances of failure observed on a very small number of B-scans. For Dataset 1, the effective range of *k* spans from 0.1 to 0.95. However, for the two subsets of Dataset 2, the valid range is slightly narrower, with the upper bound reduced to approximately 0.65. Notably, B-scans acquired with the 170 MHz antenna allow for a slightly higher upper bound. The discrepancy is likely due to the nature of the datasets. Dataset 1 was acquired in an ideal survey environment, which was an open field where there were very few small subsurface objects, resulting in minimal clutter and clearly visible hyperbolas. In contrast, Dataset 2 was collected in an urban residential area with realistic and complex disturbance, as well as multilayer pavement and inhomogeneous backfill, leading to more clutter and generally weaker hyperbolas. Additionally, due to its internal physical properties, the low-frequency antenna in Dataset 2 is less sensitive to small objects, which contributes to lower clutter compared to the higher-frequency antenna. Although the presence of interference narrows the valid range of *k*, high-frequency antennas under complex urban conditions are representative of typical GPR operations. The reasonably wide effective range of *k* observed in the Dataset 2 600 MHz subset therefore demonstrates the practical robustness of the algorithm. In practice, based on the findings drawn from the results, the recommended range of the selected *k* is 0.2 to 0.55, safely distant from both bounds to ensure stable performance.

**Figure 22:**
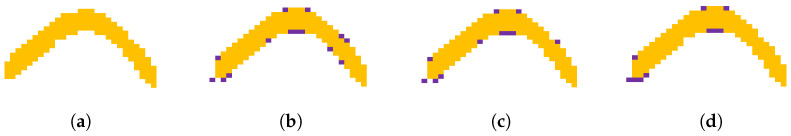
DLTS results for the example image (B-scan for a pipe with an outer diameter of 20 cm) at different window sizes: (**a**) 3; (**b**) 5; (**c**) 7; (**d**) 9. The impact of window size on the number of pixels contained in the output hyperbola is not significant, nor does it follow a clear pattern. Since the hyperbola in (**a**) contains the most pixels, in subfigures (**b**–**d**), the missing pixels included in the hyperbola compared with (**a**) are filled in purple, whereas the overlapping pixels among all subfigures are filled in yellow.

With respect to the window size, Figure 22 shows the DLTS results for window sizes of 3, 5, 7, and 9. The original B-scan in Figure 22 is of a pipe with an outer diameter of 20 cm. The extracted hyperbolas show no significant divergence from one another, with only subtle differences of a few pixels. However, the hyperbola at a window size of 3 is slightly larger than the others. The average runtime of 1000 executions correlates with the window size, with values of 6.66, 6.43, 6.46, and 6.72 milliseconds for window sizes of 3, 5, 7, and 9, respectively. This suggests that the choice of window size primarily depends on the size and resolution of the image, and its influence on the algorithm’s performance is relatively minor.

## 5. Conclusions

In response to the limitations of existing automatic techniques for segmenting the hyperbolas of subsurface pipes from GPR B-scans, especially when applied to various datasets acquired in different medium and field conditions, a novel and unsupervised model has been proposed. This model includes a ground reflection removal algorithm (GRRA), a data gravitational force enhancement (DGFE) model, and a global–local thresholding and segmentation algorithm, which features a dilation-based local thresholding and segmentation (DLTS) method.

The input B-scan is first processed through GRRA using a Fourier frequency-domain filter and a power-enhancement spatial filter, effectively removing the majority of ground reflection strips. Next, DGFE, the key component of the model, enhances the target hyperbola and distinguishes it from its multiples and the background. A global–local thresholding and segmentation model is then applied to extract the hyperbola, with DLTS performing local thresholding and segmentation simultaneously, achieving a balance between computational speed and image quality. The performance of the proposed model was further evaluated using field data from two datasets together with a few state-of-the-art techniques, where the effectiveness of the algorithms was confirmed, and the outcomes of DLTS with varying parameter values were discussed. The proposed model has been demonstrated to meet the intended objectives and holds promise for edge data processing in field GPR detection operations.

Nevertheless, as previously discussed, this work has several limitations. After extracting hyperbolas from real-world B-scans, fitting them, and calculating pipe burial depth and attributes, it was observed that the estimated burial depth was almost always greater. This discrepancy may be due to pipe settlement caused by soil compaction during backfilling, which was not considered in the experimental design. This issue makes accurate assessment of the actual performance of the algorithm challenging. Additionally, when applied to small-scale pipes with an outer diameter of 10 cm, the algorithm occasionally produces hyperbola shapes that are smaller than ideal. Finally, although the proposed method has been applied to GPR data acquired in different environmental settings, all datasets were collected using the same device. Signals obtained from different GPR platforms may exhibit distinct characteristics, and therefore, cross-device generalization remains untested. Moreover, although the urban residential area represents a relatively cluttered environment, it is not the most challenging scenario. More complex subsurface conditions, such as reinforced concrete pavement with rebar or geologically stratified backfills, are beyond the scope of this study. These limitations highlight the need for further validation of the method’s generalizability in future work.

The focus of future improvements should be on experimental design. To further validate the algorithm’s performance and provide a more solid foundation for its improvement and iteration, stricter requirements must be placed on the pipe burial process. In other words, the experimental conditions should be as consistent as possible with the real-world conditions encountered when using GPR for measurements. In the future, the experimental design should better account for pipe displacement caused during the burial process. For example, after backfilling, additional observational methods could be used to recalibrate the actual position of the pipeline. Moreover, the proposed model inevitably loses part of the hyperbolic reflection during processing, which is later restored using the raw data. This process shares certain similarities with recent studies on B-scan data completion, where missing or corrupted traces in the raw signal are reconstructed using statistical interpolation [36,37] or machine learning-based methods [38,39] to approximate the most likely signal.

## Figures and Tables

**Table 1:** Differences in SCRs and pipe depths of Datasets 1 and 2.

Data	Typical SCR	Max Depth of Pipes	Min Depth of Pipes
Dataset 1	4.934	0.20 m	0.10 m
Dataset 2 600 MHz	3.834	1.60 m	0.68 m
Dataset 2 170 MHz	3.532	1.60 m	0.68 m

**Table 2:** Statistics of runtime tests for the proposed workflow (in seconds).

Dataset	Mean	Max	Min	Median	Std. Dev.
Dataset 1	0.0685	0.112	0.0594	0.0628	0.0323
Dataset 2	0.130	0.176	0.117	0.122	0.0122

**Table 3:** Test of the algorithm flow on the real-world dataset at k=0.7 and a window size of 5.

$R_{\mathrm{actual}}$	${\bar{R}}_{\mathrm{est}}$	${\bar{\mathrm{error}}}_{R}$	$h_{\mathrm{actual}}$	${\bar{h}}_{\mathrm{est}}$	${\bar{\mathrm{error}}}_{h}$
10	9.20	8.0%	10	10.62	6.2%
7.5	6.94	7.5%	10	10.59	5.9%
5	4.26	14.8%	10	10.98	9.8%

**Table 4:** Performance statistics of the proposed model, Method A with OSCA, Method B with DCSE, and Method C with HTCA.

Method	$B_{\mathrm{shape}}$	$B_{\mathrm{connected}}$	$N_{\mathrm{cluster}}$
Proposed	36	2	0.2
Method A	28	12	0.075
Method B	39	3	24.175
Method C	39	3	27.975

**Table 5:** Average runtimes of the proposed model, Method A with OSCA, Method B with DCSE, and Method C with HTCA (in seconds).

Dataset	Proposed	Method A	Method B	Method C
Dataset 1	0.0685	0.136	$6.75\times 10^{-3}$	$8.91\times 10^{-3}$
Dataset 2	0.130	0.109	$4.33\times 10^{-3}$	$8.60\times 10^{-3}$

## Data Availability

The datasets used in this study were obtained from geological surveys conducted in real environments and from a subsurface investigation in a residential urban area. These data contain geophysical measurements and information on critical underground infrastructure, which are subject to confidentiality requirements at both the institutional and project levels. Consequently, the datasets cannot be made publicly available. As an alternative, the acquisition parameters of the GPR device are provided in Table A1 and Table A2 in Appendix A to facilitate the collection of similar data and the reproduction of the results reported in this work.

## Abbreviations

The following abbreviations are used in this manuscript:

GPR	Ground-penetrating radar
GRRA	Ground reflection removal algorithm
DGFE	Data gravitational force enhancement
DLTS	Dilation-based local thresholding and segmentation
DFT	Discrete Fourier transform
SCR	Signal-to-clutter ratio
OSCA	Open-scan clustering algorithm
C3	Column connection clustering algorithm
HTCA	Hyperbolic trend clustering algorithm

## Appendix A

### Appendix A.1. Configurations for Collecting Dataset 1

The GPR operation configurations when collecting Dataset 1 are listed in Table A1.

**Table A1:** Configurations for collecting Dataset 1.

Field	Value	Unit
Data bit width	16	N/A
Antenna center frequency	600	MHz
Number of samples	500	N/A
Number of stacks	1	N/A
Time window	65.104	ns
Distance interval	0.018812	m

### Appendix A.2. Configurations for Collecting Dataset 2

The GPR operation configurations when collecting Dataset 2 are listed in Table A2.

**Table A2:** Configurations for collecting Dataset 2.

Field	Value	Unit
Data bit width	32	N/A
Antenna center frequency	600 and 170	MHz
Number of samples	600	N/A
Number of stacks	1	N/A
Time window	78.125	ns
Distance interval	0.018325	m
